# Generation of Individual Diversity: A Too Neglected Fundamental Property of Adaptive Immune System

**DOI:** 10.3389/fimmu.2014.00208

**Published:** 2014-05-13

**Authors:** Eric Muraille

**Affiliations:** ^1^Laboratoire de Parasitologie, Faculté de Médecine, Université Libre de Bruxelles, Brussels, Belgium

**Keywords:** adaptive immune system, immune repertoire, major histocompatibility complex, individual diversity, convergent evolution, group selection

## Abstract

The fitness gains resulting from development of the adaptive immune system (AIS) during evolution are still the subject of hot debate. A large random repertoire of antigenic receptors is costly to develop and could be the source of autoimmune reactions. And yet, despite their drawbacks, AIS-like systems seem to have been independently acquired in several phyla of metazoans with very different anatomies, longevities, and lifestyles. This article is a speculative attempt to explore the selective pressures, which favored this striking convergent evolution. It is well known that the AIS enables an organism to produce a specific immune response against all natural or artificial antigenic structures. However, it is frequently neglected that this response is highly variable among individuals. In practice, each individual possesses a “private” adaptive immune repertoire. This individualization of immune defenses implies that invasion and escape immune mechanisms developed by pathogens will certainly not always be successful as the specific targets and organization of the immune response are somewhat unpredictable. In a population, where individuals display heterogeneous immune responses to infection, the probability that a pathogen is able to infect all individuals could be reduced compared to a homogeneous population. This suggests that the individual diversity of the immune repertoire is not a by-product of the AIS but of its fundamental properties and could be in part responsible for repeated selection and conservation of the AIS during metazoan evolution. The capacity of the AIS to improve the management of cooperative or parasitic symbiotic relationships at the individual level could be a secondary development due to its progressive integration into the innate immune system. This hypothesis constitutes a new scenario for AIS emergence and explains the selection of MHC restriction and MHC diversification.

## Introduction

The properties of the adaptive immune system (AIS) have always fascinated immunologists. Through the random combination of genes that breaks the central dogma of DNA conservation, the AIS is theoretically able to generate a virtually unlimited number of antigenic receptors. This anticipatory repertoire allows for (i) specific interaction with an infinite variety of microorganisms, (ii) development of long-term protective specific memory, and (iii) partial transfer of specific immune memory to the next generation. Until recently, the AIS has been presented as the pinnacle of metazoan immune system evolution, a privilege of jawed vertebrates. The AIS has traditionally been opposed to the phylogenetically conserved innate immune system (IIS), long considered as an archaic and stereotyped mechanism to control infectious microorganisms.

Development of the AIS is associated with significant energetic costs (1, 2) and autoimmune risks (3), which thus require sizeable fitness gains to justify its selection and conservation during evolution. Paradoxically, despite knowledge on its organization that has been accumulated for almost a century, the nature of those supposed fitness gains are still far from clear.

## The “Jawed Vertebrate Lifestyle” Hypothesis

Numerous authors have suggested that selective pressures leading to the emergence of the jawed vertebrate AIS are linked with greater longevity (4), the acquisition of a jaw as a result of increased physical injury in the digestive tract (5) or the necessity to protect a limited number of offspring from infection-related losses (6). However, all of these hypotheses, which are closely tied with the jawed vertebrate lifestyle have lost pertinence since the publication of studies demonstrating that functional and unrelated AIS-like systems are also present in jawless vertebrates [Variable Lymphocyte Receptors (VLR)] (7) and arthropods such as *Drosophila* [Down syndrome cell adhesion molecule (Dscam)] (8). Recently, several other unrelated gene families have also emerged as strong candidates responsible for the generation of an alternative AIS: the fibrinogen-related proteins (FREPs) (9) of the gastropod *Biomphalaria glabrata*, the variable domain-containing chitin-binding proteins (VCBPs) (10) of the amphioxus *Branchiostoma floridae* and the Sp185/333 gene family found in sea urchin (11). Even if the precise implications of these mechanisms in the host defense against infection remain unclear, the growing body of data in favor of the existence of AIS-like systems in jawless vertebrate and arthropods suggests that the paradigm of the AIS viewed as “a privilege of jawed vertebrates” is dead.

The extraordinary convergent evolution toward a similar “AIS solution” (i.e., anticipative and random generation of a large repertoire of antigenic receptors) in several phyla of metazoans seems to exclude that historical contingencies have played a major role in its selection. As phyla possessing an AIS display very different anatomies, longevities, and lifestyles, the “AIS solution” seems to constitute a response to fundamental selective pressures to which the majority of higher metazoans are subjected. The main problem is to identify these selective pressures.

## The “Superior Protection Against Infection” Hypothesis

As noted by Hedrick (12), the majority of hypotheses regarding emergence of the AIS are built on the idea that the AIS necessarily confers better individual protection against pathogens. However, this presumption brings up three fundamental problems:

(1) From a theoretical evolutionary point of view, if hosts and parasites are engaged in an “arms race” [Red Queen race metaphor of Van Valen (13)], each new defense mechanism would induce the selection of a new escape strategy. And indeed, numerous pathogens are perfectly able to manipulate the modern AIS to escape the immune response and persist in their host throughout its life (14, 15). The high genomic plasticity of virus and bacteria frequently allows them to rapidly escape new immune defense mechanisms. The fact that AIS deficiencies are frequently fatal in mice and humans is generally cited to prove the importance of the AIS in individual protection (16). But this can also be explained by the fact that infectious agents have co-evolved with hosts and consequently progressively “calibrated” their virulence to the presence of the AIS (12).
(2) Under natural conditions, all organisms are always infected by pathogens, suggesting that health is not synonymous with the “absence of infection” but results from the “good management of infection.” In some cases, tolerance can be more economic and less damaging for the host compared with the development of a sterilizing immune response (17).
(3) Infections have several positive consequences on the evolution/adaptation of organisms and their individual fitness. For example, they allow for the circulation of genetic innovation by horizontal gene transfer (HGT), help maintain genetic diversity within a population and in some cases enhance host resistance to infection [discussed in Ref. (18)], suggesting that complete neutralization of infection by the immune system can be unfavorable to long-term host adaptation.

In summary, there would be no “perfect definitive solution” to control infection at the individual level as pathogens can always neutralize a specific defense mechanism through their higher rate of genetic variation. When infectious microorganisms induce low damage level, tolerance can constitute a better economic compromise as the immune response can be highly damageable and chronic infections can have also some positive consequences. Thus, we cannot formally exclude the “superior protection against infection” hypothesis to explain selection and stabilization of the AIS. However, as noted by numerous authors (12, 19–21), it remains difficult to explain how the more specific immune response generated by the AIS improves the defense of an individual against pathogens. In other terms, why was a new complex system, based on a new strategy, required in some metazoans? Why not just have continued to gradually adapt the IIS based on germline-encoded receptors to contain infectious microorganisms?

## The “Management of Microbiota” Hypothesis

Based on the increasingly prevalent view that microbes are an essential part of the metazoan phenotype and strongly influence the fitness of their host, several authors (20–22) have proposed that emergence of the AIS in vertebrates has been in part favored by an improved ability to regulate the cooperative (mutualistic) microbial flora (called microbiota). However, members of Cnidaria, the most primitive phylum of metazoans, such as Hydra, already display a specific microbiota that is tightly regulated by their IIS [reviewed in Ref. (23)]. This association with a specific, complex, dynamic, and regulated microbiota appears to be a characteristic shared by all metazoans, which suggests that microbiota have constituted a major selective pressure that has driven evolution of the IIS. However, though acquisition of the AIS provided for more dynamic control of microbiota, direct selection of the primordial AIS mainly mediated by microbiota seems improbable. Once again, what was the need for a completely different system to perform a task previously attributed to the ISS? Why not just continue to improve and adapt the IIS?

## A New Perspective on Selection of the Adaptive Immune System: The “Generator of Individual Diversity” Hypothesis

“I stand upon my desk to remind myself that we must constantly look at things in a different way,” John Keating from Dead Poets Society.

Theories presented thus far to explain the emergence of the AIS seem unconvincing, unsatisfactory or incomplete. Without excluding these hypotheses, it is maybe time to explore AIS emergence from a new perspective.

The modern mammal AIS could theoretically generate an enormous diversity of antigenic receptors: for example, more than 10^15^ αβ T-cell receptors (TCR) (24) could be formed. It is well known to all that this large adaptive immune repertoire produces a specific immune response against all natural or artificial antigenic structures. However, even if this phenomenon has long been described (25), it is frequently neglected that this response takes different forms among individuals.

The real size of adaptive immune repertoire in an organism is difficult to determine. However, it is clear that it is several orders of magnitude smaller than 10^18^. For example, empirical analysis of T-cell receptor β-chain diversity reveals approximately 10^6^ different clones in peripheral human blood (26, 27) and in the mouse spleen (28). In human blood, TCR β-chains associate with at least 25 different α-chains, suggesting that αβ TCR diversity ranges from 10^7^ to 10^8^ (27). As each individual repertoire is randomly generated, all members of a population display a unique “private” repertoire of antigenic receptors (29, 30).

In addition to mechanisms generating a random combination of genes, it is now well documented that the natural adaptive repertoire is also largely influenced by self-dominant antigens and microbiota. Natural IgM and IgG autoantibodies are abundant and ubiquitous in the serum of mice and humans (31). These autoantibodies are generally polyreactive and can be protective against some pathogens (32). Microbiota can shape the repertoire of peripheral lymphocytes (33) and also protect the host through “competition- and immune-mediated colonization protection” (34). In turn, initial individual variations in the AIS can affect reactivity to self-antigens and microbiota. Consequently, each individual harbors a private repertoire of self-specific (35) and microbiota-specific (33) antibodies.

When TCRβ sequences among individual mice of the same inbred strain were analyzed, only 20–25% of the sequences were shared (29). Thus, even if TCR specificity is degenerate (36), we can postulate that the ability of the AIS to specifically recognize and react to a pathogen varies at the individual level.

**Figure 1:**
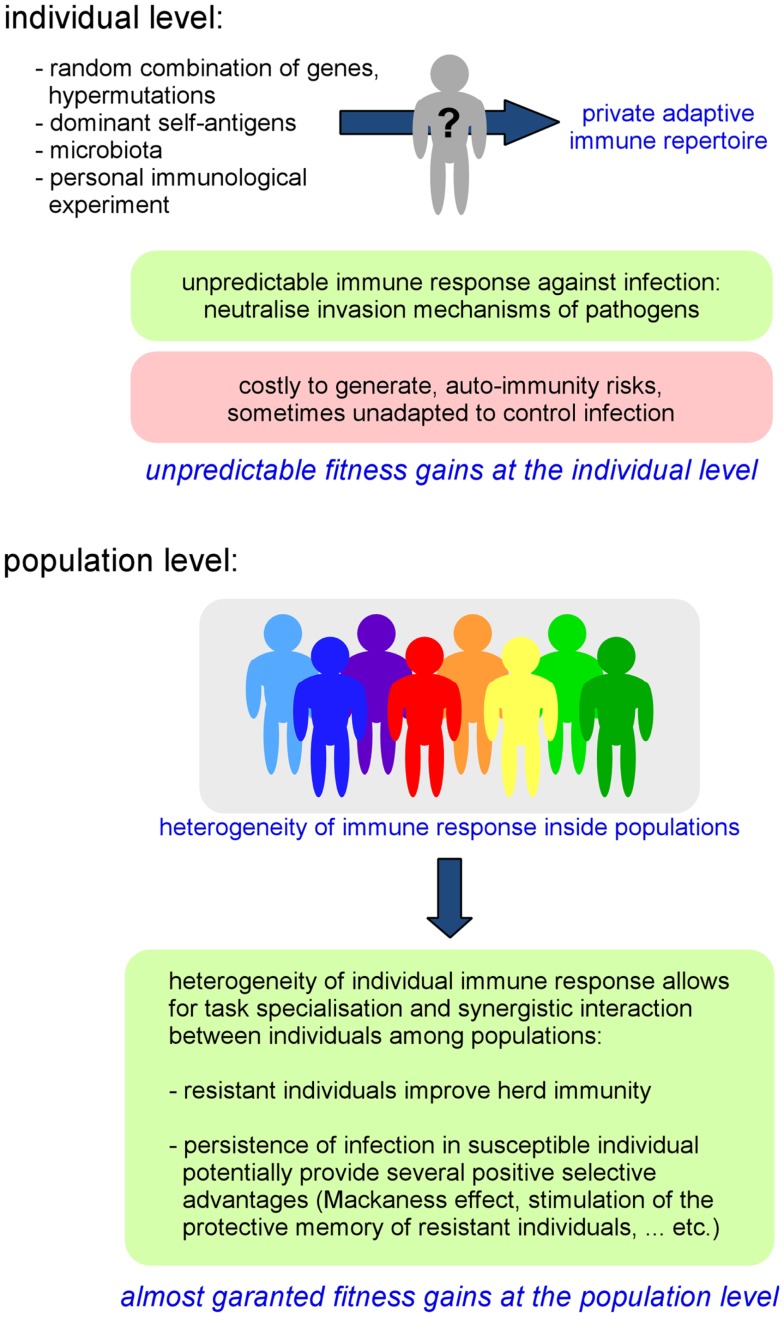
**Fitness gains at the individual and population level generated by the AIS-induced private immune repertoire**.

As a direct consequence of individual variability in the adaptive repertoire, the global immune response against pathogens can be highly heterogeneous, even within a genetically homogeneous population (37, 38). The importance of the pre-immune repertoire on the ability to control infection has been demonstrated in several experimental models, such as in *Leishmania major* infection. In this model, T-cell tolerance to a single antigen, the *Leishmania* homolog of receptors for activated C kinase (LACK), is able to drastically affect the choice of T-helper differentiation and the outcome of infection (39). It is interesting to remark that the specificity of AIS memory can also contribute to reinforce phenotypic diversity within populations. Each individual displays a particular infectious and immune history. An individual’s immune history can modify its reaction to infection, thus reinforcing random repertoire-induced individual variability. Moreover, as AIS components such as IgA are critically implicated in the composition of microbiota (40), we cannot exclude that individual variability of immune repertoires could also influence the composition of microbiota and thus potentially affect all microbiota-dependent functions such as resistance to infection (34), nutrition, metabolism (41, 42), and even behavior (43, 44). Thus, we can conclude that the AIS acts as a “generator of individual phenotypic diversity” inside populations (Figure 1).

On the whole, the property of the AIS to generate individual diversity leads to the individualization of immune defenses and implies that invasion and escape immune mechanisms developed by pathogens will certainly not always be successful as the specific targets and organization of the immune response are somewhat unpredictable/chaotic. In a population, where individuals display heterogeneous immune responses toward infection, the probability that a pathogen is able to infect all individuals could be reduced compared to a homogeneous population. This increased frequency of resistant individuals can increase the herd immunity threshold of that population (45) (i.e., the obstacle formed by resistant individuals, which limits the dissemination of an infection and thus indirectly protects susceptible individuals). Thus, individual resistance to infection could be (indirectly) favored by the heterogeneity of the population (Figure 1).

Interestingly, the heterogeneity of immune responses within a population also favors the coexistence of both “naturally immune” and “susceptible” individuals in that same population. The interest of naturally resistant individuals is evident as they confer robustness to infection at the population level. However, susceptible individuals, which allow for the persistence of pathogens inside a population [termed “super-spreaders” (46), i.e., the small number of individuals in any population that control most transmission events] can also potentially provide several positive selective advantages associated with infection [i.e., gene circulation by HGT, conservation of genetic diversity within the population (18)]. In addition, when a partially immune colonized population encounters an uncolonized population, the immune status of experienced individuals constitutes a potent competitive weapon against non-immune individuals (47–49). Finally, the persistence of pathogens at low levels can also protect hosts by stimulating the “Mackaness effect” (50), a form of immune-mediated colonization protection (34) and help to stimulate and maintain the protective memory of resistant individuals.

We can interpret the random generation of diversity by the AIS as a mechanism that favors task specialization of individuals and thus a form of cooperation between individuals of a population. Each individual displays a particular immune response toward infection and thus each individual plays a different role (a continuum between resistant and susceptible) during epidemics. As discussed above, both resistance and susceptibility can favor herd immunity. The main constraint affecting all cooperative behaviors is their potential exploitation by selfish individuals (also called cheaters) [Reviewed in Ref. (51)]. To generate durable fitness gains for stabilization and dissemination within a population, cooperative mechanisms must be protected against cheaters. In the case of the AIS, the risk of exploitation is neutralized by the random nature of generation of the individual immune repertoire. A selfish individual cannot choose to express the adapted repertoire (associated with resistance to infection) and ameliorate its fitness because the selected genetic process does not allow this choice. More importantly, it is also impossible for an individual to anticipatively know which repertoire is adapted or unadapted. AIS-dependent individual fitness gains seem to be conferred by a “lottery-like assignment,” a classical strategy to protect cooperative systems from exploitation by selfish individuals that is well described in social unicellular organisms such as *Dictyostelium discoideum* (51). I hypothesize that the random nature of the mechanisms shared by all AIS to generate individual phenotype diversity has been selected during evolution in part because of its resistance to cheating.

## Hypothetical Scenario of AIS Emergence Satisfying the Occam’s Razor Principle

The “Big Bang” vision (52) of emergence of the AIS postulates that the primordial AIS developed as a solution to improve individual survival. This vision leads to a paradox. It is very difficult to imagine the gradual building of the AIS in a Red Queen arms race dynamic because genes implicated in the modern AIS are strongly interdependent. To confer an advantage on the host and be selected, the AIS must appear as an organized set of interacting genes, and this constitutes a highly improbable stochastic event. In contrast, the “generator of individual diversity” hypothesis follows the principle of parsimony (Occam’s razor) and allows us to imagine the gradual building of the AIS without improbable big bang-like emergence.

To generate individual phenotypic variation inside populations and be selectable, the primordial AIS may have initially appeared as a “simple” mechanism generating random diversity in the expression levels or splicing of pattern recognition receptors (PRRs) or even basic receptors mediating pathogen invasion. The immunoglobulin superfamily (IgSF), which includes antigen receptors and MHC molecules, is a large group of soluble cell surface proteins. IgSF are involved in a variety of cellular activities, including tissue organization and immune responses. Many pathogens have exploited cell surface IgSF proteins to mediate cell host attachment (53, 54). An individual variation in the expression of these receptors may have immediate positive effects that are selectable at the population level. In agreement with this scenario, recombination activating genes (RAGs) have been detected in Echinoderms (55), suggesting that RAGs were present in vertebrate genomes long time before the appearance of modern antigen receptors. We can hypothesize that RAGs primarily induced simple individual variability in the expression of IgSF proteins implicated in host infection by some pathogens. This mechanism could lead to enhanced heterogeneity of populations composed of individual expressing functional RAGs bound to IgSF proteins. This heterogeneity may have improved the survival of these populations during competition with infectious organisms and populations displaying lower heterogeneity. Then, duplication of IgSF genes may have been selected because it led to a progressive increase in individual phenotypic diversity. Subsequently, this core mechanism could have gradually associated and integrated with the IIS to better manage infection and microbiota, like in the modern AIS.

## Selection of MHC Restriction

In mammals, antigen receptors on B-cells and γδT-cells recognize conformational epitopes on native antigenic proteins and glycolipids. In contrast, lymphocytes expressing αβTCR only recognize linear peptide fragments associated with the major histocompatibility (MHC) receptors. Some MHC genes are the most variable functional genes in the vertebrate genome and among the most studied regions in the human genomes. It is generally proposed that the primary function of the MHC is “*to allow the immune system to identify infectious pathogens and eliminate them*” (56). However, the selection during evolution of “MHC restriction” that results in a hole in the immune repertoire is difficult to explain from an individual selection perspective because susceptibility to infectious disease (57, 58) and a great number of immunological pathologies, such as hypersensibility (59) and autoimmunity (60), are linked to the MHC haplotype. In contrast, if the random constitution of a large antigenic receptor repertoire was initially selected for its property to favor individual phenotypic diversity, we can interpret the selection of MHC restriction for αβTCR during evolution and the later intense diversification of the MHC gene family. Functionally, MHC receptors form a filter influencing antigen perception by αβTCR and thus strongly shape the individual αβTCR repertoire during thymic selection. The high allelic polymorphism of MHC genes among populations further reinforces the wide variety of pre-immune individual repertoires and guarantees great phenotypic diversity at the population level. In keeping with this observation, analyses of the constant region have shown that γδTCR sequences are older (61) than αβTCR and B-cell receptors. This suggests that the ancestral “primordial” immune lymphoid cell was like a modern γδT-cell performing direct antigen recognition and that secretion of antigenic receptors or MHC restriction are derived characteristics. In accord, a recent report (62) confirmed experimentally that MHC restriction is the result of thymic selection and not an intrinsic feature of αβTCR structure. Interestingly, the possible additional role of the MHC in social recognition and mating choice in mammals to maintain genetic diversity (63) fits perfectly with this hypothesis.

## Conclusion

There is much evidence from ecological studies demonstrating that the ability of populations to adapt, maintain their productivity, and survive to infection is favored by the individual diversity of the components of those populations, in part through task specialization and synergic interactions between individuals (64–67). The analysis of artificial networks and living systems has formally demonstrated that a system must be robust against environmental perturbations in order to evolve and that robustness grows with the diversity of the components of the system (68). Since the variety of environmental perturbations can potentially be unlimited, natural selection should logically try to maximize the internal phenotypic diversity of populations.

The existence of individual phenotype diversification mechanisms is well documented for pathogens. Numerous pathogens escape the immune response by their ability to quickly diversify and evolve in the host. For example, the high error rates of replication in RNA viruses, such as HIV and the hepatitis C virus, cause virus populations to form mutant clouds (also termed quasispecies). It has been long thought that growth competition only occurs between different variants and that the fittest clones pre-dominate under given conditions. However, recent studies have provided evidence that complex cooperative and interfering interactions also take place within mutant clouds (69, 70). Similarly, bacterial persistence in a host is frequently associated with cooperative processed leading to biofilm formation. Biofilm impairs some immune effector mechanisms (71) and can constitute a hot spot of gene exchange (72). Like for the virus cloud, resistance to immune selective pressures in the biofilm is favored by cooperation between individuals and random genetic diversification.

The extreme variability between individual repertoires of adaptive immune receptors is well known from Oudin’s seminal work on idiotypy in the rabbit in 1969 (25). However, while individual variability is considered by geneticists, ecologists, and microbiologists as a fundamental requirement to adapt and resist to fluctuating and unpredictable selective pressures, most experimental immunologists still seem to consider variation of the individual immune response only as a by-product of the AIS maturation process and as disturbing noise in experiments. Experimental immunologists have mainly focused their work on elucidation of the molecular mechanisms implicated in the building of adaptive immune repertoires and have very rarely investigated the possibility that individual variability in and of itself may confer a fitness gain.

I propose that evolution could have selected individual phenotype diversification mechanisms in metazoans to better resist to epidemic threats, which constitute a pre-dominant selective pressure (73). Programed random genetic variations in somatic cells implicated in the AIS influence the ability of the immune system to control pathogens. This generates a large heterogeneity of individual phenotypes within a population without affecting the germline cells. I propose that this phenotypic heterogeneity allows for a large range of unpredictable answers to biotic environmental challenges and consequently results in enhanced robustness of the population. Thus, like sexuality that was presumably selected to favor gene mixing, the repeated emergence, and conservation of an AIS among metazoans can constitute one of the answers to the absolute necessity to maintain and generate phenotypic individual diversity in populations. Consequently, I hypothesize that the ability to induce random variation in the individual immune response may have constituted an important fitness gain contributing to selection and stabilization of the primordial AIS. Of course, the modern mammal AIS is clearly a highly complex multitasking system. My hypothesis concerning the fitness gains resulting from the generation of individual diversity does not exclude that other benefits, such as optimization of the management of infection and microbiota, may have also contributed to AIS selection.

I hope that my hypothesis can lead immunologists and ecologists to explore the importance of AIS-generated individual immune system diversity and collect data to confirm or invalidate this prediction.

## Theoretical Predictions

A theoretical scientific framework must satisfy Popperian’s refutability criteria by presenting testable predictions. In the field of evolutionary biology, this task is particularly difficult. Natural evolutionary processes appear extremely complex because they depend on many variables and, under laboratory conditions; they are generally difficult or impossible to reproduce. In this article, I have tried to explore the possibility that the ability of the AIS to generate individual diversity within a population constitutes one cause for its repeated selection during evolution. I conclude that individual diversity induced by the modern AIS could confer important selective advantages for individual and population survival. These advantages imply that the generation of diversity may not be a by-product of the modern AIS but of its fundamental properties. This suggests that gains resulting from the generation of diversity could be partially responsible for the repeated selection and conservation of the primordial AIS during metazoan evolution. I propose two testable predictions of the importance of AIS-induced anticipated phenotypic diversification to improve fitness and survival:

### The metazoan AIS must be based on anticipative and random generation of the antigen receptor repertoire to be selected during evolution

The adaptive immune response to pathogens does not necessarily require the anticipative random generation of a large repertoire of antigen receptors. The CRISPR/CAS system expressed by some bacteria also allows for an adaptive immune response, memory, and transfer of protection to descendants (74). This “quasi Lamarckian” system demonstrates that an alternative mechanical solution exists to generate an AIS. Why all are described metazoan AIS based on anticipatory random generation of a large repertoire of antigen receptors? This solution is costly (1) and a potential source of autoimmunity (3). Based on my hypothesis, this solution generates stable individual phenotypic diversity within populations, which offers some important advantages such as (i) introduction of unpredictability of the individual immune response, thus neutralizing the immune escape strategy of pathogens, (ii) improvement of herd immunity by favoring task specialization and cooperation among populations, (iii) protection of AIS-generated cooperation against cheating by cheaters. Discovery and characterization of new forms of the AIS among metazoans could confirm or invalidate this prediction of the constraints that govern the metazoan AIS organization.

### Reduction of MHC diversity in a population must negatively affect resistance to epidemia threats

If the metazoan AIS has been selected in part for its ability to generate individual diversity inside a given population and if this property has been conserved in the modern AIS, the uniformization of individual immune responses should reduce the global resistance of populations to epidemics. The impact of the reduction of global genetic diversity inside a group on resistance to infection has been documented in nature (75). Unfortunately, the specific contribution of AIS-induced diversity to this susceptibility is rarely investigated. As discussed previously, the combination of MHC receptors expressed by individuals strongly shapes the T-cell repertoire and the high polymorphism of the MHC locus in populations contributes to the heterogeneity of the individual immune repertoire. Thus, the variety of pre-immune individual repertoires can be a direct function of the diversity of MHC genes among populations. And the diversity of MHC genes can be a function of the prevalence of infection in a population. As a result, a reduction of MHC diversity in individuals or a population should reduce the diversity of the individual immune repertoire in the population and thus the resistance of that population to infection. In agreement, some studies [reviewed in Ref. (56, 58)] document the preservation of MHC diversity in populations by infectious diseases. MHC heterozygosity has enhanced the resistance of individuals to infection (76, 77). Certain species with reduced MHC diversity, such as the cotton-top tamarin, are more susceptible to viral infection (Piekarczyk MS JI 1997) or, like Tasmanian devils, display frequent population crashes (78).

## Conflict of Interest Statement

The author declares that the research was conducted in the absence of any commercial or financial relationships that could be construed as a potential conflict of interest.
